# Supplementary material for: Quality of life and its associated factors among epileptic patients attending public hospitals in North Wollo Zone, Northeast Ethiopia: A cross-sectional study
Source: PLoS One. 2021 Feb 23;16(2):e0247336. doi: 10.1371/journal.pone.0247336 (PMC7901738; doi:10.1371/journal.pone.0247336)
Supplement: Attachment — Submitted filename: Manuscript.docx [file pone.0247336.s002.docx]

# Abstract

**Background**: Epilepsy is one of the most common neurological diseases globally. Around 80% of epileptic patients live in developing countries and many are suffering from frequent uncontrolled seizure attacks. Besides, epilepsy is thought to be caused by witchcraft, evil spirit and God’s punishment for sins in many developing countries. As a result people with epilepsy and their families usually suffer from stigma, discrimination, depression and other psychiatric problems. Thus, this study aimed to assess the quality of life and its associated factors among epileptic patients attending public hospitals in north Wollo Zone, northeast Ethiopia.

**Methods**: An institution based cross-sectional study was employed in this study. Health related quality of life was measured based on the total score of Quality Of Life in Epilepsy inventory (QOLIE-31). A simple random sampling technique was employed. Data were entered into Epi-data 3.1 statistical package and exported to SPSS Version 20 for further analysis. Linear regression models were used to assess the relationship between quality of life and the independent variables. P-value of less than 0.05 was taken as a cut-off point to declare a statistically significant association between independent variables and the quality of life.

**Results:** A total of 395 patients participated in the study making the response rate 98.5%. The mean age of the participants was 32.39 ±30 years. More than half, 60.4% of them were females. The overall weighted average health related quality of life score was 86.54±24.309. More than half, 199 (50.4%) of epileptic patients have overall weighted average health related quality of life score above or equal to the mean score level. Male sex, higher educational status, and age at onset of epilepsy were positive predictors for health related quality of life among epileptic patients. On the other hand, having family history of epilepsy treatment follow-up duration, having uncontrolled seizure, pre-treatment number of seizures, poor adherence to anti-epileptic drugs, having moderate anxiety and depression, having sever anxiety and depression, believing that epilepsy is caused by evil spirit, drinking alcohol, and having other co morbidities were negative predictors of health related quality of life among epileptic patients.

**Conclusions:** Almost half of the epileptic patients have an overall weighted average health related quality of life score below the mean score level. Furthermore, multiple variables including family history, uncontrolled seizure, poor adherence, and having anxiety and depression were associated with health related quality of life among epileptic patients. Hence, targeting these variables in epilepsy management is recommended.

**Keywords:** Quality Of Life; Epilepsy; Associated factors.

## Background

Epilepsy is a neurological disorder and is characterized by recurrent seizures episodes involving part of the body or the entire body. Seizures episodes might sometimes be followed by loss of bowel, and bladder control and compromised consciousness (1). Globally, more than 50 million people live with epilepsy with an annual cumulative incidence rate of 67.77 per 100,000 persons (2, 3).

Many factors including stroke, brain tumor, head injury, central nervous system infections, and genetic risks are believed to be the cause of epilepsy. Different types of antiepileptic drugs are available, and are highly effective for the treatment of Epilepsy. Approximately 70% of epileptic patients are estimated to successfully respond for proper treatment with antiepileptic drugs (AED) (2-4).

Despite the fact that most epileptic patients can become free from seizure attacks with the optimal use of AEDS, the treatment outcome in the majority of cases remains insufficient and many suffer from uncontrolled seizure attacks especially in low-income countries (5). Treatment outcome of epilepsy is associated with several factors. Gender of epileptic patients, age of the patient at seizure onset, type of epileptic seizure, seizure frequency, etiology of epilepsy, and presence of co-morbidities are among these factors (6-8).

Unlike developed countries, people in developing countries especially in Africa, don’t have enough knowledge about epilepsy and is thought by many to be caused by witchcraft, evil spirit and God’s punishment for sins and they even don’t seek medical care when they are seek. As a result, epileptic patients and their families usually suffer from stigma, discrimination and prejudice (2, 9).

It is shown that, patients with epilepsy and their families most of the time prefer traditional and religious healers and in house prayers (9). Similarly, the self-management practice of epileptic patients is very low. In relation to this, studies revealed that in developing countries, the rate of antiepileptic drug therapy adherence is very poor (9, 10).

As a result, not only from recurrent seizure attacks, but also patients with epilepsy are suffering from impaired quality of life (QOL). QOL is a broad idea and includes the person’s physical health, mental health, the level of independence, social health, personal beliefs and their relationship to each other and with the outside environment (11).

There is a tremendous negative impact caused by the poor adherence, low self-management practice, stigma and other co morbid conditions on patient’s QOL, especially concerning to the working conditions and personal and social relationships (12). Therefore, the psychosocial problems including anxiety, depression, social stigma, and deficiency of social backing and unemployment can adversely affect their QOL (13-16)**.**

Moreover, when patients took an AED therapy for a period of time, that drug can provoke adverse effects which can further impair their QOL (4, 17). For these reasons, identifying predictors of reduced QOL in epileptic patient’s is critical to improve interventions and management strategies for epilepsy (18). Such growing recognition of the importance of the psychosocial effects of epilepsy has led to the need to quantify QOL in affected individuals with epilepsy (16, 19).

Ethiopia shares the burden of epilepsy and over 1 million people are estimated to have active epilepsy (20). The community’s awareness about epilepsy in Ethiopia is not different from other African countries. As a result, epilepsy is believed to be caused by witchcraft, evil spirit, and God's punishment for sins by many (21, 22). Consequently, patients with epilepsy in Ethiopia are suffering from physical disability, stigma, discrimination and other psychiatric problems (10). Despite this devastating impact, the public health community does not give epilepsy the attention it deserves. Hence, this study aimed to assess quality of life and the factors which are associated with it among patients on anti-epileptic drug therapy in north Wollo Zone public hospitals.

# Methods

## Study setting

This study was carried out in north Wollo zone public hospitals, Amhara region, Ethiopia. According to the 2007 Census conducted by the Central Statistical Agency of Ethiopia (CSA), North Wollo Zone has a total population of 1,500,303, of whom 752,895 are men and 747,408 women; with an area of 12,172.50 square kilometers. The largest ethnic group in north Wollo is the Amhara (99.38%) and all other ethnic groups made up 0.62% of the population. Amharic is spoken as a first language by 99.28% of the population and the remaining 0.72% spoke other primary languages. 82.74% of the populations are Ethiopian Orthodox Christianity followers, and 17.08% are Muslims(23). There are 5 public hospitals in this zone; namely Kobo primary hospital, Lalibela primary hospital, Meket primary hospital, Wadila primary hospital and Woldia Comprehensive specialized hospital.

## Study design and period

An institution based cross-sectional study was conducted from February, 01to June 30, 2020.

### Source population

Adult patients (age ≥ 18 years), who have been diagnosed with epilepsy, and are on at least one anti-epileptic drug therapy in north Wollo zone public hospitals.

### Study population

Adult patients (age ≥ 18 years), who have been diagnosed with epilepsy and have been on regular follow- up for at least one year with at least one anti-epileptic drug therapy in north Wollo zone public hospitals.

## Eligibility criteria

All adult epileptic patients (age ≥ 18 years), who have been on regular follow- up for at least one year with at least one anti-epileptic drug therapy were included. Because, seizure freedom 1 year after AED treatment is suggested as a good predictor of long-term remission(24).Epileptic patients who are mentally incapable, have psychiatric problems and are incapable of delivering proper information, those who are seriously ill and unresponsive for the interview, and those who have hearing problems were excluded.

**Sample size determination**

The sample size for this study was determined by using the single population proportion formula considering the assumptions: The proportion of epileptic patients having good quality of life, 38.6%, taken from a study in Addis Ababa (p = 0.386) (25). Level of significance 5% (α = 0.05), Z α/2 =1.96 and margin of error 5% (d = 0.05). The sample size was calculated as follows:

**n_o_	=	Z (α/2)^2^*****p (1-p)**

**d^2^**

Hence, the minimum required sample size was = $(1.96)2*(0.386) (0.614)/ (0.05)2$= **364.** Adding 10% non-response rate, the final sample size appeared to be **401**.

## Sampling Technique and Procedure

Simple random sampling technique was employed to select the required number of epileptic patients attending public hospitals in north Wollo zone. First, three representative hospitals were selected randomly. Then, in these selected hospitals, the number of study participants were assigned proportionally based on the number of epileptic patients in each of those hospitals.

# Operational definitions

**Quality of life** is defined as good if a participant scores mean and above the mean score of quality of life related questions and, poor if the score is below the mean score of quality of life measuring questions.

**Seizure control**: The seizure status was considered to be controlled if the patient had not experienced any seizure attacks in the last one year and not-controlled if the patient experienced one or more seizure attacks in the last one year follow up period.

**Anxiety and depression**: The four-item patient health questionnaire for anxiety and depression (PHQ-4) was used to measure anxiety and depression among epileptic patients. The total score was determined by adding together the scores of each of the 4 items. Scores were rated as normal (0-2), mild (3-5), moderate (6-8), and severe (9-12) (26).

**Adherence**: Adherence to AED treatments was measured using the four-item Morisky Medication Adherence Scale (MMAS-4) (27). Each item is scored as either 0 (Yes) or 1 (No). The score of each item is then summed to give a range of scores from 0 to 4. A score of 3 - 4 is considered that the patient is adherent and a score of ≤ 2 is considered not adherent (28).

## Data Collection Technique and Tools

A structured and pretested interviewer-administered, Amharic version questionnaire was used to collect data from the study participants. The questionnaire was adapted from previous comparable studies and was prepared in English. Then, it was translated to Amharic and back to English for consistency of the questions.

The questionnaire has three sections. The first section was about socio demographic characteristics of the participants. The second section covers a data extraction checklist on seizure control status and related issues. The third section consists the Quality Of Life in Epilepsy (QOLIE)-31questionner which is a survey question of health-related quality of life for adults (18 years or older) with epilepsy. Health related quality of life was measured based on total score of QOLIE-31questionner. A QOLIE- 31questionner consists 31 items categorized under seven domains covering the following concepts of health: Overall Quality of Life (2 items), Medication Effects (3 items), Energy Fatigue (4 items), Seizure Worry (5 items), Emotional Well-being (5 items), Cognitive Function (6 items), and Social Function (5 items). The raw scores are rescaled from zero to 100 with higher values reflecting better QOL. Each item is scored on a scale of 0 to 100, with a score of zero representing worst quality of life and a score of 100 representing a higher quality of life. However, possible response sets for scoring vary across questions. Examples of response sets used include (i) 0, 25, 50, 75, 100; (ii) 0, 20, 40, 60, 80, 100; and (iii) 0, 33, 67, 100 (29).

## Data Quality Assurance

To ensure the quality of data, a one day training was given for the supervisors and data collectors on; the objectives of the study, the questions and extent of explanations, the way to keep privacy and confidentiality and other ethical issues. The supervisors will coordinate the overall data collection process. Pre-test study was conducted on 5% of the sampled population by taking 23 epileptic patients at Dessie referral hospital which is not included in the study settings. The pre-test study was conducted 2 weeks before the actual data collection period. The study subjects who fulfill the inclusion criteria were selected by simple random sampling technique. The result of the pretest was discussed and all the necessary amendments were made on, the instructions, contents, order, and grammatical issues based on the pretest results. All data were checked for completeness, accuracy, clarity and consistency by the supervisors and the investigators each night after the data were collected. Investigators and the supervisors closely monitored the data collection process. Double data entry and validation were performed and data were intensively cleaned before analysis.

## Data Processing and Analysis

Data were coded and entered into Epi-data version 3.1statistical program and then exported to SPSS Version 20 for further analysis. Descriptive statistical methods were computed for the study variables. A simple linear regression model was used to assess the relationship between quality of life and the independent variables and then, a multiple regression analysis was run to assess the association between independent variables while controlling confounders. P-value of less than 0.05 was taken as a cut-off point to declare statistically significant association between independent and dependent variables.

**Results**

**Sociodemographic and clinical characteristics of respondents**

A total of three hundred ninety five (395) participants volunteered and provided a complete response for the interview making the response rate 98.5%. The mean age of the participants was 32.39 and the median was 30 years. Two hundred seventy four (60.4%) of them were females. Majority, that is 255 (64.6%) of the participants were orthodox Christianity followers followed by 130	(32.9%). Regarding marital status, 187 (47.3%) were married, 146 (37.0%) single, 49 (12.4%) divorced and the remaining 13 (3.3%) were widowed. More than half, 223 (56.5%) of the participants were rural dwellers.

Regarding education, 203 (51.4%) of the participants have no formal education and the remaining 49.6% have completed up to higher levels of education. Occupationally, 133 (33.7%) of the participants were farmers, 52	(13.2%) government employee, 40 (10.1%) merchant, 79 (20.0%) student, 25 (6.3) daily laborers and 66 (16.7%) others (house wife, unemployed). Around 60 percent of the participants have generalized type of seizure and the remaining had partial type of seizures (Table 1).

**Table 1: Sociodemographic and clinical characteristics of epileptic patients, 2020 (𝑛 = 395).**

| **Characteristics** | **Category** | | **Frequency** | **Percentage** | |
| --- | --- | --- | --- | --- | --- |
| Age, years | |  |  | |  |
| Mean±SD | | (32.39±10.71) |  | |  |
| Sex | |  |  | |  |
|  |  | Male | 231 | | 58.5 |
|  |  | Female | 164 | | 41.5 |
| Religion | |  |  | |  |
|  |  | Orthodox | 255 | | 64.6 |
|  |  | Muslim | 130 | | 32.9 |
|  | | Protestant | 10 | | 2.5 |
| Marital status | |  |  | |  |
|  |  | Married | 187 | | 47.3 |
|  |  | Single | 146 | | 37.0 |
|  |  | Divorced | 49 | | 12.4 |
|  |  | Widowed | 13 | | 3.3 |
| Residence | |  |  | |  |
|  |  | Urban | 172 | | 43.5 |
|  |  | Rural | 223 | | 56.5 |
| Educational status | |  |  | |  |
|  | | No education | 203 | | 51.4 |
|  |  | Primary | 74 | | 18.7 |
|  |  | Secondary | 66 | | 16.7 |
|  |  | Higher | 52 | | 13.2 |
| Age at onset of epilepsy (years) | |  |  | |  |
|  |  | <30 | 300 | | 75.9 |
|  |  | 30-45 | 78 | | 19.7 |
|  |  | >45 | 17 | | 4.3 |
| Duration of epilepsy follow up (years) | |  |  | |  |
|  |  | <=2 years | 107 | | 27.1 |
|  |  | > 2 years | 288 | | 72.9 |
| Epilepsy type | | Generalized | 236 | | 59.7 |
|  |  | Partial | 159 | | 40.3 |
| Pre-treatment duration with epilepsy | |  |  | |  |
|  |  | ≤ 12 month | 249 | | 63.0 |
|  |  | >12 months | 146 | | 37.0 |
| Pre-treatment number of seizures | |  |  | |  |
|  |  | ≤ 5 seizure | 125 | | 31.6 |
|  |  | > 5 seizures | 270 | | 68.4 |
| Treatment regimen | |  |  | |  |
|  |  | Monotherapy | 163 | | 41.3 |
|  | | Polytherapy | 232 | | 58.7 |

**Health related quality of life among epileptic patients**

The average total quality of life score of the seven domains of health related quality of life among epileptic patients was as follows: Seizure worry (63.43±26.56); Overall quality of life (58.48±21.22); Emotional wellbeing (62.73±18.34), Energy/fatigue (58.00±20.48), Cognitive function (73.03±23.01), Medication effects (71.49±25.62), and Social functioning (74.97±22.56). The overall weighted average quality of life score was 86.54±24.309. More than half, 199 (50.4%) of epileptic patients have overall weighted average quality of life score above or equal to the mean score level, and the remaining,196 (49.6%) have overall weighted average quality of life score below the mean.

**Factors associated with health related quality of life**

In the bivariate linear regression analysis; participants age, educational status, family history of epilepsy, epilepsy treatment follow-up duration, having uncontrolled seizure, pre-treatment number of seizure, poor adherence to anti-epileptic drugs, having sever anxiety and depression, poor sleep pattern, believing that epilepsy is caused by evil spirit, drinking alcohol, cigarette smoking, chewing Chat and having other co morbidities were significantly associated with the health related quality of life among epileptic patients. On the multivariable linear regression analysis model, about 50.8% of the total variation in health related quality of life among epileptic patients was explained by the variables included in the model. Male sex (B _=_ 4.34, 95%CI: 0.41, 8.27, P_=_ 0.03), higher educational status (B _=_ 7.18, 95%CI: 1.39, 13.00, P _=_ 0.015), and age at onset of epilepsy (B _=_ 0.237, 955CI:0.017, 0.45, P _=_ 0.035), were associated with increased health quality of life score.

On the other hand, having family history of epilepsy (B _=_ -4.78,95%CI: -9.24,-0.33, P = 0.035), epilepsy treatment follow-up duration, having uncontrolled seizure (B _=_ -11.08, 95% CI:-15.11,-7.05, P _=_ 0.000), pre-treatment number of seizures (B _=_ -4.86, 95%CI:-8.91,-0.81, P _=_ 0.019), poor adherence to anti-epileptic drugs (B _=_ -11.65, 95%CI: -16.06,-7.23, P _=_ 0.000), having moderate anxiety and depression (B _=_ -4.526, 95%CI: -8.59, -0.46, P _=_ 0.029), having sever anxiety and depression (B = -12.84, 95CI: -18.30,-7.37, P _=_ 0.000), believing that epilepsy is caused by evil spirit (B _=_ -7.04, 95%CI: -11.46,	-2.61, P _=_ 0.002), drinking alcohol (B _=_ -5.42,95%CI:-10.72,-0.13, P _=_ 0.045), and having other co morbidities (B _=_ -9.35, 95%CI: -14.35,-4.36, P < 0.001) were significantly associated with the health related quality of life among epileptic patients (Table 2).

Table 2: Linear regression analysis of factors for health related quality of life in epileptic patients, 2020 (𝑛 = 395).

| **Independent**  **Variables** | **Crude**  **Beta** | **95% CI for**  **Beta** | **P-value** | **Adjusted Beta** | **95% CI for**  **Beta** | **P-value** |
| --- | --- | --- | --- | --- | --- | --- |
| Age |  |  |  |  |  |  |
| Mean±SD (32.39±10.71) | -0.29 | (-0.51,-0.07) | 0.011 | -0.252 | (-0.53,0.02) | 0.072 |
| Sex |  |  |  |  |  |  |
| Male | 3.55 | (-1.33,8.42) | 0.153 | **4.34** | **(0.41,8.27)** | **0.030** |
| Female | 1 |  |  |  |  |  |
| Educational status |  |  |  |  |  |  |
| No education | 1 |  |  |  |  |  |
| Primary | 1.02 | (-5.15,7.19) | 0.75 | 2.91 | (-2.23,8.06) | 0.267 |
| Secondary | 2.75 | (-3.70,9.19) | 0.403 | 3.05 | (-2.60,8.69) | 0.289 |
| Higher | 5.41 | (-1.70,12.51) | 0.135 | **7.18** | **(1.39,13.00)** | **0.015** |
| Marital status |  |  |  |  |  |  |
| Married | 7.16 | (2.39,11.93) | 0.003 | 7.21 | (-2.99,17.42) | 0.165 |
| Single | 1.21 | (-3.78,6.19) | 0.635 | 0.81 | (-9.97,11.60) | 0.882 |
| Divorced | -14.83 | (-21.99-7.68) | <0.001 | -3.19 | (-14.34,7.96) | 0.574 |
| Widowed | 1 |  |  |  |  |  |
| Family history of epilepsy |  |  |  |  |  |  |
| No |  |  |  |  |  |  |
| Yes | -9.44 | (-15.25,-3.64) | 0.002 | **-4.78** | **(-9.24,-0.33)** | **0.035** |
| Age at onset of epilepsy |  |  |  |  |  |  |
| Mean±SD (24.62±12.58) | 0.15 | (-0.05,0.34) | 0.138 | **0.24** | **(0.02,0.46)** | **0.035** |
| Epilepsy follow up (years) |  |  |  |  |  |  |
| Mean±SD(5.20±4.20) | 0-.99 | (-1.56,-.43) | .001 | -0.12 | (-0.60,0.36) | 0.624 |
| Uncontrolled seizure | -20.49 | (-24.89.-16.10) | <0.001 | **-11.08** | **(-15.11 -7.05)** | **<0.001** |
| Epilepsy type |  |  |  |  |  |  |
| Generalized | 4.03 | (-0.86,8.93) | 0.106 | -1.62 | (-5.39,2.14) | 0.397 |
| Partial | 1 |  |  |  |  |  |
| Pre-treatment number of seizure |  |  |  |  |  |  |
| >=5times | -7.270 | (-12.40,-2.14) | 0.006 | **-4.86** | **(-8.91,-0.81)** | **0.019** |
| < 5times | 1 |  |  |  |  |  |
| Treatment regimen |  |  |  |  |  |  |
| Monotherapy | 1 |  |  |  |  |  |
| Polytherapy | 3.64 | (-1.242,8.51) | 0.144 | -0.72 | (-4.44,3.00) | 0.703 |
| Shortage of AEDs | 0.32 | (-4.68,5.32) | 0.900 | -2.21 | (-6.07,1.65) | 0.260 |
| Adherence to AEDs |  |  |  |  |  |  |
| Good | 1 |  |  |  |  |  |
| Poor | -25.86 | (-29.97,-21.76) | <0.001 | **-11.65** | **(-16.06,-7.23)** | **<0.001** |
| Anxiety and depression |  |  |  |  |  |  |
| Mild | 1 |  |  |  |  |  |
| Moderate | 0.66 | (-4.28,5.61) | 0.792 | **-4.53** | **(-8.59,-0.46)** | **0.029** |
| Severe | -25.35 | (-30.99,-19.72) | <0.001 | **-12.84** | **(-18.30,-7.37)** | **<0.001** |
| Sleep pattern |  |  |  |  |  |  |
| Poor | 9.66 | (4.78,14.55) | <0.001 | 2.97 | -0.10,6.94) | 0.142 |
| Good | 1 |  |  |  |  |  |
| Belief that epilepsy is due to evil spirit |  |  |  |  |  |  |
| Yes | -16.80 | (-22.23,-11.38) | <0.001 | **-7.04** | **(-11.46,-2.61)** | **0.002** |
| No | 1 |  |  |  |  |  |
| Alcohol drinking |  |  |  |  |  |  |
| No | 1 |  |  |  |  |  |
| Yes | -14.50 | (-20.26,-8.74) | <0.001 | **-5.42** | **(-10.72,-0.13)** | **0.045** |
| Cigarette smoking |  |  |  |  |  |  |
| No | 1 |  |  |  |  |  |
| Yes | -10.73 | -20.96,-0.50) | 0.040 | 5.13 | (-4.37,14.63) | 0.289 |
| Chat chewing |  |  |  |  |  |  |
| No | 1 |  |  |  |  |  |
| Yes | -7.96 | (-15.50,-0.42) | 0.039 | -3.10 | (-10.60,4.40) | 0.417 |
| Co morbidity |  |  |  |  |  |  |
| No |  |  |  |  |  |  |
| Yes | -16.31 | (-22.30 ,-10.32) | <0.001 | **-9.35** | **(-14.35 -4.36)** | **<0.001** |

**Discussion**

In this study, around half, 49.6% of epileptic patients had poor quality of life. This finding is consistent with study findings conducted at (49.7%) Jimma University and (45.8%) Addis Ababa at Amanuel specialized hospital respectively, (30, 31). It is also in line with a study conducted at University of Gondar Referral Hospital which has reported that 44.2% of participants were found having poor quality of life (32). A study in Italia has reported a consistent finding that 43.3% of patients had poor quality of life at a health institution study (33).

However, it is higher than a study at Ambo General Hospital that 24.4% had overall poor health related quality of life (34). This variation might be due to differences in the tool and operational definitions. Unlike, the current study, the previous study has used the World Health Organization (WHOQOL-BREF) tool for measuring the health related quality of life among epileptic patients and the current study was conducted using the Quality of Life Inventory for Epilepsy (QOLIE-31).

Regarding the quality of life score of the seven domains of QOLIE-31, participants scored the lowest average score on the Energy/fatigue domain, and the highest score was found in the Social functioning domain. A similar study has reported a consistent finding that participants have scored the highest mean under social functioning domain (31). This means patients with epilepsy are having an acceptable social interaction with the community which is encouraging. However, the low score under energy/fatigue domain is worrying because it is in indication that patients with epilepsy are suffering from tiredness, loss of energy and related impacts on their life (34).

Regarding the factors related with quality of life, male sex, attaining higher education, and ages at the onset of epilepsy were positively associated with quality of life among epileptic patients. Male epileptic patients had a higher quality of life compared to female epileptic patients. This finding is in line with a study in Portugal that female gender was associated with a poorer health related in patients with refractory epilepsy (35). A consistent finding was also reported in India that female gender was associated with reduced quality of life among epileptic patients(36). This might be due to the effect of hormonal changes occurring throughout a woman's life which can influence and be influenced by seizure mechanisms and AEDs, presenting unique management challenges and this in turn may affect their quality of life (37). It might be also due to lack of social support. Unlike men, women in Ethiopia usually stay at home and work in house and might not get social support from the community.

Epileptic patients who attended higher education had higher quality of life compared to those not attended education. This finding is supported by studies conducted at Ambo general hospital (34), Mekelle City, Northern Ethiopia (38), Uganda (39), Kenya (40), and Iraq (41). A consistent finding was also obtained in Indonesia whereby level of education was one of the predictors of quality of life in patients with epilepsy(42). This could be due to the influence of education on individual perception of their disease condition and adherence to their medications. It might be also due to employment opportunities because people with higher educational status are highly likely to have different job offers and be employed by both governmental and non-governmental organizations.

As the age of onset of epilepsy increased, the quality of life of patients also increased. This is supported by a study conducted in Malaysia (43). It is likely that a person with epilepsy diagnosed in childhood would have many schooling hours disrupted if the frequency of seizures were uncontrolled and this could also lead to low educational achievement and thus a lower employability potential. Furthermore, a seizure itself may cause accidents and injuries thus preventing sufferers from driving and depriving many types of employment opportunities, this in turn has the potential of negatively affecting the quality of life of epileptic patients (44).

On the other hand, the present study revealed that having family history of epilepsy, increased treatment follow-up duration, having uncontrolled seizure, pre-treatment number of seizures, poor adherence to anti-epileptic drugs, believing that epilepsy is caused by evil spirit, drinking alcohol, moderate to severe anxiety and depression and having other co morbidities were negative predictors of quality of life among epileptic patients.

Family history of epilepsy was associated with decreased the quality of life among epileptic patients. This is supported by a study conducted among children and adolescents in Iran which has reported that family history of epilepsy was significantly associated with health related quality of life (45). A consistent finding was also found in a study conducted among patients with refractory mesial temporal lobe epilepsy in Brazil (46) . This might be due to the influence of perceived stigma among epileptic patients. Because, when there is epilepsy in a family, the community might have stigmatizing behavior towards the family as epilepsy is believed to be caused by God’s curse, evil spirit and witchcraft by many (47). Furthermore, people might see epilepsy as contagious diseases, which in turn may result in lack of social support, stigma and discrimination among patients (48, 49).

The present study showed pre-treatment number of seizures, and having uncontrolled seizure negatively affects the quality of life of epileptic patients. The finding is supported by studies conducted in Ethiopia (38, 50), Uganda (39), and Iraq (41). The possible reason could be because of seizure frequency, a known risk factor which cause excessive fear, inability to work, stigmatization, diminishing hope and future life, impairment in social functions, and psychological impairments (43). Individuals with frequent epileptic seizures will always be in discomfort, and they do not know when the next seizure will happen, as a result they took care and impose restrictions from driving, cooking, and doing risky jobs to avoid having seizure episodes at inappropriate times, places, or social events (51, 52). These in turn may compromise the quality of life of the patients.

Moderate to severe anxiety and depression ,and having other co morbidities results in decreased quality of life of epileptic patients which is supported by studies conducted in China (53), Ethiopia (32, 50), and Kenya (54). This could be due to depression affected quality of life particularly on the domains touching on pain, lethargy/fatigue, and emotional well-being (54) Believing that epilepsy is caused by evil spirit negatively affects the quality of life of epileptic patients. This could be due to the influence of cultural beliefs on attitudes and uncaring actions of the community towards patients with epilepsy (55) which in turn can result in high rates of low self-esteem in patients with epilepsy, thus adversely affecting quality of life (56).

Furthermore, increased treatment follow-up duration, and drinking alcohol negatively affects quality of life of epileptic patients. This might be due to the effect of alcohol on the frequency of seizure attacks resulting in possible negative effect on quality of life. Because, This might be due to the fact that alcohol is a known precipitating factor for seizure disorders (57, 58). Furthermore, alcohol consumers might have interaction of antiepileptic drugs with the alcohol and might further result in recurrent seizure attacks among patients (59, 60). This might be also due to poor adherence to medications as a result of prolonged treatment for epilepsy with AEDs. Poor drug adherence is a known factor which is found to have a negative association with health quality of life among epileptic patients in this and many others studies, and, might result in poor quality of life if patients are non-adherent to AEDs.

# Conclusion

Almost half of epileptic patients have an overall weighted average health related quality of life score below the mean score level. Furthermore, multiple variables including family history, uncontrolled seizure, poor adherence, and having anxiety and depression were associated with health related quality of life among epileptic patients. As a result, targeting these variables in epilepsy management is essential. Moreover, early recognition and addressing poor adherence, and detecting and managing co-morbid conditions by the clinician will have a great impact on controlling seizure attacks and increasing quality of life of epileptic patients.

# Declaration

## **Ethical approval and consent to participant**

Ethical clearance was obtained from Woldia University institutional review board and support letters were written to the selected public hospitals and concerned others to obtain permission and cooperation during the data collection process. Written informed consent was obtained from each study participant prior to the data collection process. All participants were asked for their willingness to participate in the study and were told that it will not have any risk on them. Confidentiality of the information was preserved and privacy of the respondents was maintained by making the questionnaires anonymous and putting in secured places after data were collected.

## Abbreviations

AED: Anti-Epileptic Drug; LMICs: Low and Middle Income Countries; PWE: People Living With Epilepsy; SPSS: Software Package of Social Science; WHO: World Health Organization.

Consent for publication

Not applicable.

## Availability of data and materials

Data supporting the conclusions of this article are included within the article and its supporting files.

# Funding

The study was funded by Woldia University

Competing Interests

The authors have declared that no competing interests exist.

## Author’s Contribution

AMK participates in all steps of the study from its commencement to writing. BBA, MWK, AD, AW, and MA have participated in reviewing the paper, analysis and interpretation of the data. All authors have read and approved the submission of the final manuscript.

# Acknowledgments

The authors would like to express our gratitude to the data collectors and supervisors for their great contribution. Our deepest gratitude also goes to those who participated in this study and Woldia University for its financial support.

# References

1.	Lee GP, Clason CL. Classification of seizure disorders and syndromes, and neuropsychological impairment in adults with epilepsy. Textbook of clinical neuropsychology. 2008:437-65.

2.	World Health Organization (WHO) epilepsy report; Key facts of epilepsy: . February 8, 2018, Available at <http://www.who.int/>.

3.	Ngugi AK, Kariuki S, Bottomley C, Kleinschmidt I, Sander J, Newton C. Incidence of epilepsy A systematic review and meta-analysis. Neurology. 2011;77(10):1005-12.

4.	Hwaid AH. Knowledge and awareness of papillomavirus and cervical cancer among college students and health care workers women in Diyala, Iraq. Am J Public Health Res. 2013;1(8):221-5.

5.	Laxer KD, Trinka E, Hirsch LJ, Cendes F, Langfitt J, Delanty N, et al. The consequences of refractory epilepsy and its treatment. Epilepsy & behavior. 2014;37:59-70.

6.	MekonenTefera G, Woldehaimanot TE, Tarekegn M. Poor treatment outcomes and associated factors among epileptic patients at Ambo Hospital, Ethiopia. EUROPEAN JOURNAL OF THERAPEUTICS. 2015;21(1):9-16.

7.	Shen C, Du Y, Lu R, Zhang Y, Jin B, Ding Y, et al. Factors predictive of late remission in a cohort of Chinese patients with newly diagnosed epilepsy. Seizure. 2016;37:20-4.

8.	Mohanraj R, Brodie MJ. Early predictors of outcome in newly diagnosed epilepsy. Seizure. 2013;22(5):333-44.

9.	Mutanana N, Mutara G. Health seeking behaviours of people with epilepsy in a rural community of Zimbabwe. International Journal. 2015;87.

10.	Hasiso TY, Desse TA. Adherence to treatment and factors affecting adherence of Epileptic patients at Yirgalem General Hospital, Southern Ethiopia: A prospective cross-sectional study. PloS one. 2016;11(9):e0163040.

11.	Strauss AL, Glaser BG. Chronic illness and the quality of life: Mosby St. Louis; 1975.

12.	Hopker CdC, Berberian AP, Massi G, Willig MH, Tonocchi R, editors. The individual with epilepsy: perceptions about the disease and implications on quality of life. CoDAS; 2017: SciELO Brasil.

13.	Gholami A, Salarilak S, Lotfabadi P, Kiani F, Rajabi A, Mansori K, et al. Quality of life in epileptic patients compared with healthy people. Medical journal of the Islamic Republic of Iran. 2016;30:388.

14.	Mahrer-Imhof R, Jaggi S, Bonomo A, Hediger H, Eggenschwiler P, Krämer G, et al. Quality of life in adult patients with epilepsy and their family members. Seizure. 2013;22(2):128-35.

15.	Saadi A, Patenaude B, Nirola DK, Deki S, Tshering L, Clark S, et al. Quality of life in epilepsy in Bhutan. Seizure. 2016;39:44-8.

16.	Alsaadi T, Kassie S, El Hammasi K, Shahrour TM, Shakra M, Turkawi L, et al. Potential factors impacting health-related quality of life among patients with epilepsy: results from the United Arab Emirates. Seizure. 2017;53:13-7.

17.	Nunes VD, Sawyer L, Neilson J, Sarri G, Cross JH. Diagnosis and management of the epilepsies in adults and children: summary of updated NICE guidance. Bmj. 2012;344:e281.

18.	Bashir MBA, Cumber SN. The quality of life and inequalities in health services for epilepsy treatment among patience in the urban cities of Sudan. The Pan African Medical Journal. 2019;33.

19.	Lee S-J, Kim J-E, Seo J-G, Cho YW, Lee J-J, Moon H-J, et al. Predictors of quality of life and their interrelations in Korean people with epilepsy: a MEPSY study. Seizure. 2014;23(9):762-8.

20.	Worku D. Review Article: Epilepsy in Ethiopia (P03.127). Neurology. 2013;80(7 Supplement):P03.127-P03.

21.	Henok A, Lamaro T. Knowledge about and attitude towards epilepsy among Menit Community, Southwest Ethiopia. Ethiopian journal of health sciences. 2017;27(1):47-58.

22.	Fekadu W, Mekonen T, Bitew S, Mekonnen TC, Menberu M, Shewangizaw S. Community’s Perception and Attitude towards People with Epilepsy in Ethiopia. Behavioural neurology. 2019;2019.

23.	Federal Democratic Republic of Ethiopia Population census commission: Summary and Statistical report of the 2007 Population and Housing Census. 2008, Addis Ababa: UNFPA, 9-10.

24.	Lindsten H, Stenlund H, Forsgren L. Remission of seizures in a population‐based adult cohort with a newly diagnosed unprovoked epileptic seizure. Epilepsia. 2001;42(8):1025-30.

25.	Beyene MG, Engidawork E. Adherence and treatment outcome among epileptic patients of follow-up at Amanuel Specialized Mental Hospital, Ethiopia. Ethiopian Pharmaceutical Journal. 2017;33(1):53-64.

26.	Kroenke K, Spitzer RL, Williams JB, Löwe B. An ultra-brief screening scale for anxiety and depression: the PHQ–4. Psychosomatics. 2009;50(6):613-21.

27.	Morisky DE, Green LW, Levine DM. Concurrent and predictive validity of a self-reported measure of medication adherence. Medical care. 1986:67-74.

28.	Gurumurthy R, Chanda K, Sarma G. An evaluation of factors affecting adherence to antiepileptic drugs in patients with epilepsy: a cross-sectional study. Singapore medical journal. 2017;58(2):98.

29.	Devinsky O, Cramer J. the QOLIE Development Group. Professional Postgraduate Services, a division of Physicians World Communications Group, Secaucus, NJ, USA. 1993.

30.	Tegegne MT, Muluneh NY, Wochamo TT, Awoke AA, Mossie TB, Yesigat MA. Assessment of quality of life and associated factors among people with epilepsy attending at Amanuel Mental Specialized Hospital, Addis Ababa, Ethiopia. Science Journal of Public Health. 2014;2(5):378-83.

31.	Shiferaw D, Hailu E. Quality of Life Assessment among Adult Epileptic Patients Taking Follow Up Care at Jimma University Medical Center, Jimma, South West Ethiopia: Using Quality of Life in Epilepsy Inventory-31instrument. 2018.

32.	Muche EA, Ayalew MB, Abdela OA. Assessment of Quality of Life of Epileptic Patients in Ethiopia. International Journal of Chronic Diseases. 2020;2020.

33.	Pamela D, Gisele J, Jennifer Z, Cinthia PA, David M, et al. (2018) Quality of life in patients with epilepsy. J Neurol Neurol Sci Disord 4(1): 008-0010. DOI: <http://dx.doi.org/10.17352/jnnsd.0000024>.

34.	Tefera GM, Megersa WA, Gadisa DA. Health-related quality of life and its determinants among ambulatory patients with epilepsy at Ambo General Hospital, Ethiopia: Using WHOQOL-BREF. PloS one. 2020;15(1):e0227858.

35.	Silva B, Canas-Simião H, Cordeiro S, Velosa A, Oliveira-Maia AJ, Barahona-Corrêa JB. Determinants of quality of life in patients with drug-resistant focal epilepsy. Epilepsy & Behavior. 2019;100:106525.

36.	Ashwin M, Rakesh P, Pricilla RA, Manjunath K, Jacob K, Prasad J. Determinants of quality of life among people with epilepsy attending a secondary care rural hospital in south India. Journal of neurosciences in rural practice. 2013;4(Suppl 1):S62.

37.	Stephen LJ, Harden C, Tomson T, Brodie MJ. Management of epilepsy in women. The Lancet Neurology. 2019;18(5):481-91.

38.	Gebre AK, Haylay A. Sociodemographic, clinical variables, and quality of life in patients with epilepsy in Mekelle City, Northern Ethiopia. Behavioural Neurology. 2018;2018.

39.	Nabukenya AM, Matovu JK, Wabwire-Mangen F, Wanyenze RK, Makumbi F. Health-related quality of life in epilepsy patients receiving anti-epileptic drugs at National Referral Hospitals in Uganda: a cross-sectional study. Health and quality of life outcomes. 2014;12(1):49.

40.	Kinyanjui DW, Kathuku DM, Mburu JM. Quality of life among patients living with epilepsy attending the neurology clinic at Kenyatta National Hospital, Nairobi, Kenya: a comparative study. Health and quality of life outcomes. 2013;11(1):98.

41.	Shakir M, Al-Asadi JN. Quality of life and its determinants in people with epilepsy in Basrah, Iraq. Sultan Qaboos University medical journal. 2012;12(4):449.

42.	Hawari I, Syeban Z, Lumempouw SF. Low education, more frequent of seizure, more types of therapy, and generalized seizure type decreased quality of life among epileptic patients. Medical Journal of Indonesia. 2007;16(2):101-3.

43.	Norsa’adah B, Zainab J, Knight A. The quality of life of people with epilepsy at a tertiary referral centre in Malaysia. Health and quality of life outcomes. 2013;11(1):1-6.

44.	Sillanpää M, Cross JH. The psychosocial impact of epilepsy in childhood. Epilepsy & Behavior. 2009;15(2):S5-S10.

45.	Momeni M, Ghanbari A, Bidabadi E, Yousefzadeh-Chabok S. Health-Related Quality of Life and Related Factors in Children and Adolescents With Epilepsy in Iran. Journal of Neuroscience Nursing. 2015;47(6):340-5.

46.	Pauli C, de Oliveira Thais ME, Claudino LS, Bicalho MAH, Bastos AC, Guarnieri R, et al. Predictors of quality of life in patients with refractory mesial temporal lobe epilepsy. Epilepsy & Behavior. 2012;25(2):208-13.

47.	Winkler AS, Mayer M, Schnaitmann S, Ombay M, Mathias B, Schmutzhard E, et al. Belief systems of epilepsy and attitudes toward people living with epilepsy in a rural community of northern Tanzania. Epilepsy & Behavior. 2010;19(4):596-601.

48.	Osungbade KO, Siyanbade SL. Myths, misconceptions, and misunderstandings about epilepsy in a Nigerian rural community: implications for community health interventions. Epilepsy & Behavior. 2011;21(4):425-9.

49.	Tran D-S, Odermatt P, Singphuoangphet S, Druet-Cabanac M, Preux P-M, Strobel M, et al. Epilepsy in Laos: knowledge, attitudes, and practices in the community. Epilepsy & Behavior. 2007;10(4):565-70.

50.	Abadiga M, Mosisa G, Amente T, Oluma A. Health-related quality of life and associated factors among epileptic patients on treatment follow up at public hospitals of Wollega zones, Ethiopia, 2018. BMC research notes. 2019;12(1):679.

51.	Smeets VM, van Lierop BA, Vanhoutvin JP, Aldenkamp AP, Nijhuis FJ. Epilepsy and employment: literature review. Epilepsy & behavior. 2007;10(3):354-62.

52.	Azuma H, Akechi T. Effects of psychosocial functioning, depression, seizure frequency, and employment on quality of life in patients with epilepsy. Epilepsy & Behavior. 2014;41:18-20.

53.	Wang F-L, Gu X-M, Hao B-Y, Wang S, Chen Z-J, Ding C-Y. Influence of marital status on the quality of life of Chinese adult patients with epilepsy. Chinese medical journal. 2017;130(1):83-7.

54.	Mwangala PN, Kariuki SM, Nyongesa MK, Mwangi P, Chongwo E, Newton CR, et al. Cognition, mood and quality-of-life outcomes among low literacy adults living with epilepsy in rural Kenya: A preliminary study. Epilepsy & Behavior. 2018;85:45-51.

55.	Ramasundrum V MHZ, Tan CT. Public awareness, attitudes and understanding towards epilepsy in Kelantan, Malaysia. Neurol J Southeast Asia. 2000; 5:55–60.

56.	GA B. The psychosocial burden of epilepsy. Epilepsia. 2002;43:26–30.

57.	Rathlev NK, Ulrich AS, Delanty N, D’Onofrio G. Alcohol-related seizures. The Journal of emergency medicine. 2006;31(2):157-63.

58.	Höppener R, Kuyer A, Van der Lugt P. Epilepsy and alcohol: the influence of social alcohol intake on seizures and treatment in epilepsy. Epilepsia. 1983;24(4):459-71.

59.	Vernon GM. Sex, drugs and alcohol: drug interactions of concern to consumers. 2013.

60.	Leach JP, Mohanraj R, Borland W. Alcohol and drugs in epilepsy: pathophysiology, presentation, possibilities, and prevention. Epilepsia. 2012;53:48-57.
