# Supplementary material for: Quality of life and its associated factors among epileptic patients attending public hospitals in North Wollo Zone, Northeast Ethiopia: A cross-sectional study
Source: PLoS One. 2021 Feb 23;16(2):e0247336. doi: 10.1371/journal.pone.0247336 (PMC7901738; doi:10.1371/journal.pone.0247336)
Supplement: Attachment — Submitted filename: comments plos one 2.docx [file pone.0247336.s003.docx]

The manuscript describes quality of life and its associated factors among epileptic patients attending public hospitals in North Wollo zone, Ethiopia. There are several major points that need to be addressed and revised by the authors first before accepting the manuscript for publication. Moreover, line spacing and line numbering were not applied as per the PLoS one guideline. Hence it is difficult to evaluate the manuscript point by point. The manuscript has several grammar and punctuation errors that need extensive English language revision.

**Title**

Make space “a/cross-sectional”

**Abstract**

The conclusion you made is just a summary of the result. Need revision

**Introduction**

Several studies have been conducted in Ethiopia including in Jima University, in Gondar, Ambo….What is new in your study? and please clearly state in the introduction section.

**Method**

Why at least one AED use and 1 year since diagnosis were considered as eligibility criteria?

Hoe simple random sampling was carried? How do you access the sampling frame?

You have stated “…questionnaire was adapted from previous comparable studies..” please cite some of them.

Who collects the actual data (their qualification…)?

**Result section**

“…three hundred ninety five (395)…” no need to repeat the same information

” ….Christianity followers followed by 130 (32.9%).” It is an incomplete statement and revise it.

How did you collet seizure type? (did you use medical chart review)?

The proportion of medication adherence is not included in the result section
